# Further Findings Concerning Endothelial Damage in COVID-19 Patients

**DOI:** 10.3390/biom11091368

**Published:** 2021-09-16

**Authors:** Monica Gelzo, Sara Cacciapuoti, Biagio Pinchera, Annunziata De Rosa, Gustavo Cernera, Filippo Scialò, Marika Comegna, Mauro Mormile, Gabriella Fabbrocini, Roberto Parrella, Gaetano Corso, Ivan Gentile, Giuseppe Castaldo

**Affiliations:** 1 CEINGE-Biotecnologie Avanzate, scarl, 80145 Naples, Italy; gelzo@ceinge.unina.it (M.G.); cernera@ceinge.unina.it (G.C.); filippo.scialo@unicampania.it (F.S.); marika.comegna@unina.it (M.C.); gaetano.corso@unifg.it (G.C.); 2 Dipartimento di Medicina Molecolare e Biotecnologie Mediche, Università di Napoli Federico II, 80131 Naples, Italy; 3 Dipartimento di Medicina Clinica e Chirurgia, Università di Napoli Federico II, 80131 Naples, Italy; sara.cacciapuoti@libero.it (S.C.); biapin89@virgilio.it (B.P.); mormile@unina.it (M.M.); gafabbro@unina.it (G.F.); ivan.gentile@unina.it (I.G.); 4 Dipartimento di Malattie Infettive e Emergenze Infettive, Divisione di Malattie Infettive Respiratorie, Ospedale Cotugno, AORN dei Colli, 80131 Naples, Italy; annunziataderosa@yahoo.it (A.D.R.); rob.parrella@gmail.com (R.P.); 5 Dipartimento di Medicina Traslazionale, Università della Campania L. Vanvitelli, 80131 Naples, Italy; 6 Dipartimento di Medicina Clinica e Sperimentale, Università di Foggia, 71121 Foggia, Italy

**Keywords:** E-selectin, P-selectin, MCP-2, CCL8, monocyte chemotactic protein

## Abstract

Systemic vascular damage with micro/macro-thrombosis is a typical feature of severe COVID-19. However, the pathogenesis of this damage and its predictive biomarkers remain poorly defined. For this reason, in this study, serum monocyte chemotactic protein (MCP)-2 and P- and E-selectin levels were analyzed in 204 patients with COVID-19. Serum MCP-2 and P-selectin were significantly higher in hospitalized patients compared with asymptomatic patients. Furthermore, MCP-2 increased with the WHO stage in hospitalized patients. After 1 week of hospitalization, MCP-2 levels were significantly reduced, while P-selectin increased in patients in WHO stage 3 and decreased in patients in WHO stages 5–7. Serum E-selectin was not significantly different between asymptomatic and hospitalized patients. The lower MCP-2 levels after 1 week suggest that endothelial damage triggered by monocytes occurs early in COVID-19 disease progression. MCP-2 may also predict COVID-19 severity. The increase in P-selectin levels, which further increased in mild patients and reduced in severe patients after 1 week of hospitalization, suggests that the inactive form of the protein produced by the cleavage of the active protein from the platelet membrane is present. This may be used to identify a subset of patients that would benefit from targeted therapies. The unchanged levels of E-selectin in these patients suggest that endothelial damage is less relevant.

## 1. Introduction

Endothelial activation has been reported in patients with severe COVID-19 [1]. It is triggered by inflammatory cytokines released by leukocytes and contributes to generating systemic micro- and macro-thrombosis [2]. Moreover, tissue factor [3] and platelet activation [4] also contribute. The pathogenesis of endothelial damage during severe COVID-19 remains poorly defined. A better knowledge of the molecular events involved in its pathogenesis may contribute to selecting appropriate therapeutic targets [2,5]. Necroscopies of COVID-19 patients revealed endothelial inflammation with the accumulation of lymphocytes and monocytes in the majority of important organs [6,7], particularly the lungs [8]. In addition, neutrophils, which are increased in the blood [9] and tissues [7] of COVID-19 patients, may contribute to the endothelial damage and thus the release of tumor necrosis factor (TNF)-alpha, interleukin (IL)-1, and IL-8.

Among the myriad proteins involved in inflammation, monocyte chemotactic protein (MCP)-2, also known as chemokine (C-C motif) ligand 8 (CCL8), is an innate immunity protein [10]. It acts as a chemotactic factor toward a variety of inflammatory cells, including macrophages [11], which trigger cytokine production, inflammation, and endothelial damage [2]. MCP-2 expression is strongly induced by SARS-CoV-2, cyclically reinforcing macrophage recruitment and the inflammatory response [12]. Serum levels of the protein are increased during the acute phase of COVID-19, particularly in severe patients [13].

Selectins promote platelet and leukocyte migration from vessels to tissues by helping these cells roll on the endothelial surface [14]. P-selectin is stored in platelet granules. During inflammation, the fusion of the granules with the plasma membrane helps the P-selectin become exposed on the platelet membrane. The following dimerization of the protein promotes the interaction of platelets with endothelial cells. In addition, its binding to the P-selectin ligand-1 (PSGL-1), which is expressed by leukocytes, helps leukocyte migration and the subsequent production and release of inflammatory cytokines [5]. P-selectin is also present in soluble form in serum and can be derived from alternative splicing and/or from the proteolytic cleavage of the dimeric membrane. Increased serum P-selectin levels are found in acute myocardial infarction and in other cardiovascular diseases [15]. However, it is strongly debated as to whether serum P-selectin has a biological pro-inflammatory activity or should be considered a terminal, less active form of the protein, which increases due to enhanced synthesis by platelets and endothelial cells [16,17]. This is a relevant point, as P-selectin proteolytic release from platelets and its interaction with its ligands can be modulated with currently available drugs [14,18]. Recently, serum P-selectin was defined as a diagnostic and prognostic biomarker of COVID-19 [19], despite the fact that another study reported a reduction in serum P-selectin in COVID-19 patients [20].

E-selectin is produced by various cells, including endothelial cells [21], and its expression is induced by IL-1, TNF, and IL-10 [14]. The protein is able to interact with specific receptors, which are expressed on the leukocyte surface, and to modulate the endothelial permeability to leukocytes [14]. Increased serum levels of the protein were found in a series of inflammatory and autoimmune disorders, including severe COVID-19 [22]. Furthermore, E-selectin is considered a biomarker of endothelial damage [23] even if its serum levels increase in other conditions unrelated to endothelial damage, including hypercholesterolemia, hypertension, diabetes, and cardiovascular diseases. Furthermore, various studies suggest that E-selectin has a role in endothelial regeneration rather than endothelial damage [21].

An arsenal of anti-inflammatory molecules [24] or drugs that can target the different phases of selectin-mediated chemotaxis [14] and macrophage-induced inflammation [2] are now available for patients with COVID-19. Understanding the role of these molecules in COVID-19 may help us to define personalized therapeutic approaches. For this reason, we studied the levels of P- and E-selectin and MCP-2 in serum samples from COVID-19 patients with varying severities, i.e., asymptomatic and hospitalized patients in different phases of the disease.

## 2. Materials and Methods

### 2.1. Patients

We enrolled 204 adult patients that had been diagnosed with COVID-19 (SARS-CoV-2 infection). Sixteen of these patients were not hospitalized as a result of an asymptomatic course of the disease up until a negative swab result. The remaining 188 patients were admitted from March 2020 to May 2021 to one of the following hospitals: the Department of Clinical Medicine and Surgery—Section of Infectious Diseases, University Hospital Federico II, Naples; the Department of Infectious Disease and Infectious Urgencies, Cotugno Hospital, AORN dei Colli, Naples. The study was approved by the Ethical Committee of the University Federico II of Naples (protocol code 138/20, 14 April 2020); the lone exclusion criterion was the refusal or the impossibility to obtain informed consent. The 16 asymptomatic patients had a median age of 49 years (interquartile range, IQR: 42–61) and included five females (31%). The 188 hospitalized patients had a median age of 52 years (IQR: 34–65) and included 86 females (46%). The COVID-19 diagnosis was confirmed by molecular analysis (RT-PCR) using a nasopharyngeal swab [25]. All the hospitalized patients were classified on the basis of the World Health Organization (WHO)—Research and Development Blueprint expert group’s seven ordinal scale, as used in previous influenza studies. For each patient, we considered the worst WHO stage during the infection [26,27,28].

### 2.2. Immunoassays

Serum MCP2, P-selectin, and E-selectin were measured using a Human Magnetic Luminex Assay on a Biorad Bio-Plex 100 system (Labospace srl, Milan, Italy) with a 1:2 sample dilution. The analysis was performed at diagnosis in asymptomatic patients and at hospital admission and 1 week after in hospitalized patients.

### 2.3. Statistical Analysis

Data were reported as median and IQR. The Shapiro–Wilk test was used to test the normality of distributions, the results of which were significantly non-normal. Comparisons between the two groups were evaluated using the Mann–Whitney U test. Statistical differences between the three groups were assessed using the Kruskal–Wallis test and the Mann–Whitney U test as a post-hoc test. Categorical data were reported as frequency (percentage), and the chi-square test was used to compare the frequencies. Correlations between variables were evaluated using Spearman correlation analysis. Paired comparisons were performed by Wilcoxon signed-rank test. Statistical analysis was performed using SPSS (version 26, IBM SPSS Statistics, Segrate, Italy). Graphics were produced using the KaleidaGraph software (version 4.5.4, Synergy, Reading, PA, USA). A *p*-value < 0.05 was considered significant.

## 3. Results

**Figure 1:**
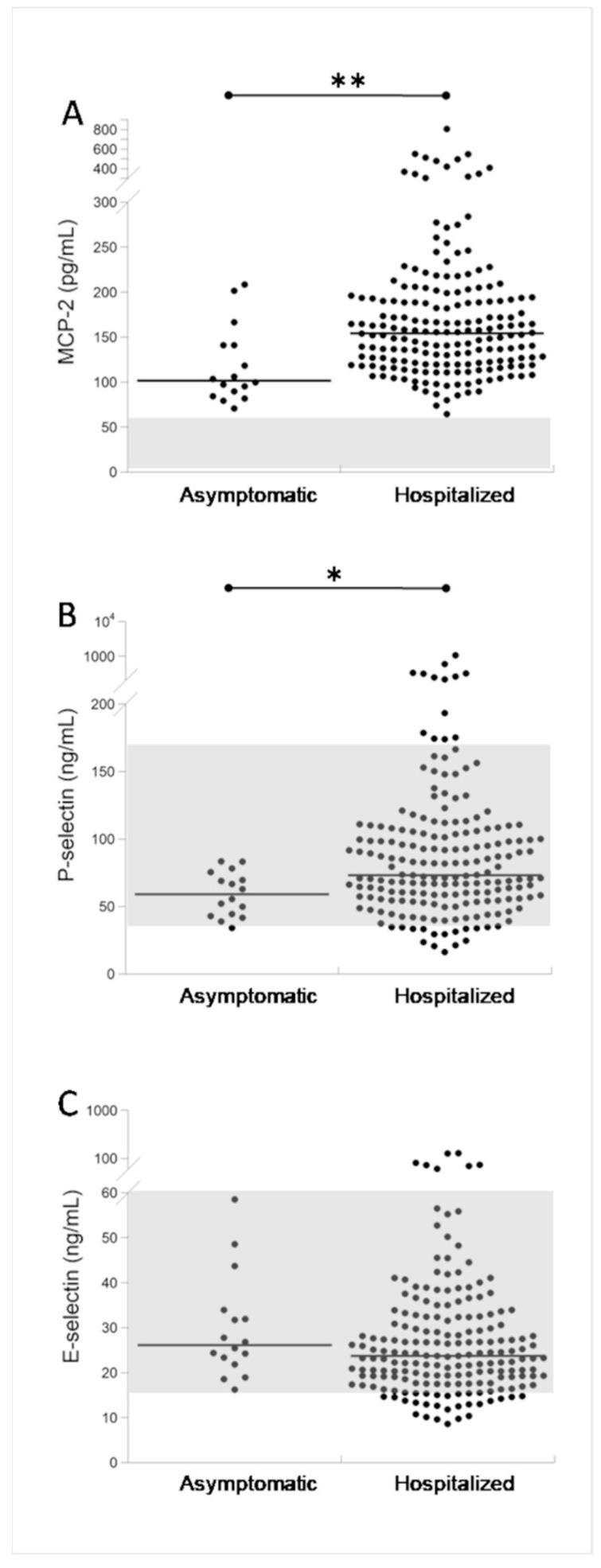
Serum MCP-2 (**A**), P-selectin (**B**), and E-selectin (**C**) in asymptomatic (n = 16) and hospitalized (n = 188) COVID-19 patients. The grey area indicates the reference intervals in healthy subjects [29,30]. * *p* < 0.01; ** *p* < 0.001.

Figure 1 shows the MCP-2, P-selectin, and E-selectin serum levels in 16 asymptomatic and in 188 hospitalized COVID-19 patients at admission. For each biomarker, the figure also shows the reference intervals in healthy subjects [29,30]. Among COVID-19 patients, all asymptomatic and hospitalized patients had higher MCP-2 levels than those in healthy subjects. Moreover, 13/188 (6.9%) hospitalized COVID-19 patients had higher P-selectin serum levels than those in healthy subjects, and again all asymptomatic patients had values within the reference range. Finally, only 7/188 (3.7%) patients had E-selectin values above the reference range, while all asymptomatic patients had values within this range. In addition, the comparison of the three biomarkers between asymptomatic and hospitalized patients showed that MCP-2 and P-selectin levels were significantly higher (*p* < 0.001) in hospitalized patients (Figure 1A,B), while the differences observed for E-selectin were not significant (Figure 1C).

Table 1 presents a comparison of the age, gender, the three serum biomarkers, platelet number, and previous existing morbidities in hospitalized COVID-19 patients classified accordingly to severity. The age and the percentage of males were significantly higher in patients in WHO stages 4 and 5–7 as compared with patients in WHO stage 3. Serum MCP-2 was significantly higher (*p* = 0.001) in patients in WHO stages 5–7 compared with patients in WHO stage 3. No significant differences were observed for serum P- and E-selectin levels or platelet number among the three groups of patients. The percentage of smokers was lower in patients in WHO stages 5–7 as compared with both WHO stage 3 and WHO stage 4. Moreover, the percentage of patients with diabetes was significantly higher in patients in WHO stages 5–7 as compared with the two other groups.

Table 2 shows a comparison of the serum levels of the three biomarkers and platelet number in COVID-19 patients (classified according to the WHO stage) at hospital admission and after 1 week of hospitalization. Serum MCP-2 levels were significantly lower after 1 week of hospitalization in all WHO subgroups of patients. Serum P-selectin was significantly (*p* = 0.003) higher in patients in WHO stage 3 after 1 week of hospitalization, while in patients in WHO stages 5–7, we observed a decreasing trend. The E-selectin serum levels were not significantly different in any of the WHO subgroups. Finally, after 1 week of hospitalization, the platelet number was only significantly (*p* = 0.003) higher in patients in WHO stage 3.

Table 3 shows the correlation of serum MCP-2, P-selectin, and E-selectin with other biomarkers. MCP-2 was positively correlated with serum P- and E-selectin and IL-10. P-selectin was positively correlated with serum E-selectin, IL-6, IL-10, and platelet number. Finally, serum E-selectin values were positively correlated with serum IL-6 and IL-10.

## 4. Discussion

In patients with COVID-19, we observed the following: (i) Enhanced serum MCP-2 levels that significantly mirrored the severity of the disease. Such an increase is transient, as after 1 week of hospitalization, MCP-2 levels significantly declined. (ii) Enhanced serum P-selectin levels, which were poorly related to the WHO severity level. The serum P-selectin further increased after 1 week of hospitalization in mild patients and declined in severe patients. (iii) No change in serum E-selectin levels.

MCP-2 is a chemotactic factor for monocytes/macrophages [31]. The increase observed in COVID-19 patients reflects macrophage activation, as the protein is mainly produced by macrophages that are stimulated by various triggers, including Sars-CoV-2 [12]. Macrophage activation is an early event in the development of COVID-19 and seems to be transient, with the exception of a few particularly severe cases, in which a cytokine storm occurs [13]. In fact, MCP-2 levels were significantly reduced after 1 week of hospitalization in most patients in our study, in agreement with a previous study [13]. Furthermore, we observed that levels of MCP-2 were significantly correlated with IL-10 levels, which is in turn produced by macrophages. Interleukin 10 is considered to be a negative modulator of inflammation; however, in COVID-19 patients, it seems to play a relevant pro-inflammatory role [32]. In fact, two studies revealed the early production of IL-10 [33] and its significant association with disease severity [34]. Thus, it is possible that either IL-10 or MCP-2 expression increases during the early phases of COVID-19 infection due to macrophage activation. Regardless, our data reveal that serum MCP-2 at hospital admission is a good predictor of COVID-19 severity. In addition, its rapid kinetics, which was also observed in a previous study [13], is useful to modulate the anti-inflammatory therapy. Interestingly, our study revealed a subset of about 20% of COVID-19 patients with particularly increased serum MCP-2 levels (>250 ng/mL). Such cases could be selected for treatment with approaches that target the macrophage-related inflammatory response [2].

Serum P-selectin seems to be a terminal inactive form with a monomeric, inactive structure obtained from the cleavage of the active protein on the platelet membrane [16]. However, the protein is produced in higher amounts during the first phase of the disease due to platelet hyper-activation [4]. This was evidenced by the higher values of serum protein observed in hospitalized COVID-19 patients as compared to control subjects, which is in agreement with previous studies [19,35]. Furthermore, serum P-selectin levels are significantly correlated with the platelet number. Thus, serum P-selectin can be considered as a biomarker of protein synthesis at the platelet level, and thus, as a marker of platelet activation. The serum protein levels were not related to the WHO stage of severity. This is again in agreement with previous studies that found that serum P-selectin values were quite similar in mild/moderate and in severe patients [19,35], thus excluding serum P-selectin as a prognostic biomarker in COVID-19 patients. The same result was previously reported in acute myocardial infarction, in which P-selectin seems to be related to the promotion of the disease, but its serum levels are not related to the severity of the disease [15]. However, we observed a significant increase in serum P-selectin (i.e., >170 ng/mL) in about 20% of COVID-19 patients, suggesting that, in a subset of patients, platelet activation is extraordinarily enhanced. These data, together with the observation that P-selectin was expressed by pneumocytes in a patient who died of severe COVID-19, suggest that a subset of COVID-19 patients may be treated with therapies that inhibit P-selectin [14,36], thus reducing severe acute lung injury, as was demonstrated in a murine model [37]. Therefore, serum protein levels may help in the selection of patients eligible for these treatments.

The highest P-selectin and MCP-2 levels, which were observed in about 20% of patients, may also depend on certain pre-existing morbidities, such as diabetes and CKD. However, the significant reduction in MCP-2 levels after 1 week of hospitalization in all three WHO subgroups suggests that the increment observed at hospital admission is mainly caused by the COVID-19 disease.

Finally, the lack of an increase in serum E-selectin in our COVID-19 patients suggests that the endothelial damage in these patients is a less relevant, secondary event caused by monocyte (as confirmed by the increase in serum MCP-2) and platelet recruitment (as confirmed by the increased P-selectin values). This is in agreement with observations from other models of inflammation, in which increases in serum P-selectin were not associated with parallel increases in serum E-selectin [38]. Moreover, our results contrast with a previous study that proposed endothelial activation as the primum movens in the pathogenesis of COVID-19, followed by platelet activation [1]. This conclusion was based on the alteration of the NO and PGI pathways observed in COVID-19 patients. Furthermore, our results are in disagreement with a previous study that reported higher serum E-selectin values in 20 severe COVID-19 patients when compared to 20 mild cases [22].

## 5. Conclusions

Our data suggest that serum MCP-2 represents an effective biomarker for COVID-19 severity and therapy monitoring as a result of its rapid kinetics. Furthermore, increased serum P-selectin (although not related to COVID-19 severity) may be used to identify a subset of patients that would benefit from targeted therapies. The lack of an increase in E-selectin in COVID-19 patients (including those in advanced stages) excludes the hypothesis that endothelial damage has a primary role in the pathogenesis of COVD-19.

## Figures and Tables

**Table 1:** Comparison of age, serum MCP-2, selectins, and platelet number in hospitalized COVID-19 patients at admission, subgrouped based on the WHO stage.

	WHO 3	WHO 4	WHO 5–7	Kruskal–Wallis
N	64	78	46	-
Females, N (%)	48 (75)	27 (35) ^a^	11 (24) ^a^	-
Age	35.5 (29.0–52.3)	55.5 (41.0–67.0) ^a^	57.5 (48.0–75.8) ^a^	<0.0001
MCP-2 (pg/mL)	133 (112–167)	158 (123–206)	172 (141–201) ^a^	0.001
P-selectin (ng/mL)	82.3 (54.3–109)	72.7 (58.3–100)	64.5 (49.2–108)	n.s.
E-selectin (ng/mL)	23.6 (17.5–30.5)	22.3 (19.0–32.4)	25.4 (18.7–32.8)	n.s.
Platelets (MN/mmc)	231 (201–301)	251 (206–361)	224 (155–257)	n.s.
Smokers, N (%)	11 (17)	13 (17)	3 (7) ^b^	-
CAD, N (%)	10 (16)	5 (7)	6 (13)	-
Diabetes, N (%)	9 (14)	8 (10)	12 (27) ^c^	-
CKD, N (%)	none	5 (7) ^a^	9 (20) ^a^	-

^a^ *p* < 0.01, versus WHO 3; ^b^ *p* < 0.01, versus both WHO 3 and WHO 4; ^c^ *p* < 0.01, versus WHO 4. CAD: coronary artery disease; CKD: chronic kidney disease; MCP-2: monocyte chemoattractant protein-2; n.s.: not significant.

**Table 2:** Comparison of serum MCP-2, selectins, and platelet number in COVID-19 patients at hospital admission and after 1 week.

	WHO 3 (n = 18)	WHO 4 (n = 24)	WHO 5–7 (n = 10)
MCP-2 (pg/mL)			
Basal	142 (111–204)	169 (122–263)	151 (125–175)
After 1 week	132 (101–160)	124 (96–158)	105 (76.7–128)
*p*-value ^a^	0.035	<0.0001	0.017
P-selectin (ng/mL)			
Basal	93.8 (66.7–124)	98.9 (74.7–135)	109 (74.6–192)
After 1 week	130 (95.1–171)	102 (95.4–127)	72.5 (57.4–165)
*p*-value ^a^	0.003	n.s.	n.s.
E-selectin (ng/mL)			
Basal	28.9 (22.6–40.7)	23.8 (19.0–38.4)	26.3 (20.0–30.0)
After 1 week	26.2 (21.9–36.5)	24.1 (17.5–39.7)	20.9 (13.8–25.1)
*p*-value ^a^	n.s.	n.s.	n.s.
Platelets (MN/mmc)			
Basal	248 (193–292)	263 (207–395)	168 (119–259)
After 1 week	339 (251–446)	319 (206–370)	229 (155–273)
*p*-value ^a^	0.003	n.s.	n.s.

^a^ Wilcoxon signed-rank test. MCP-2: monocyte chemoattractant protein-2; MN/mmc: thousands of cells/mmc; n.s.: not significant.

**Table 3:** Correlations among serum MCP-2, selectins, inflammation parameters, blood cell, and platelet number at admission.

	MCP-2 (pg/mL)		P-Selectin (ng/mL)		E-Selectin (ng/mL)	
	r_s_	*p*-Value	r_s_	*p*-Value	r_s_	*p*-Value
MCP-2 (pg/mL)	-	-	0.183	0.012	0.271	<0.0001
P-selectin (ng/mL)	0.183	0.012	-	-	0.145	0.047
E-selectin (ng/mL)	0.271	<0.001	0.145	0.047	-	-
IL-6 (pg/mL)	0.065	0.377	0.278	<0.0001	0.340	<0.0001
IL-17A (pg/mL)	−0.099	0.330	−0.077	0.452	−0.051	0.616
IL-10 (pg/mL)	0.203	0.006	0.463	<0.0001	0.347	<0.0001
Neutrophils (N/mmc)	0.004	0.954	−0.012	0.867	0.046	0.527
Monocytes (N/mmc)	0.113	0.121	0.082	0.262	0.099	0.178
Platelets (N/mmc)	−0.039	0.691	0.340	<0.0001	−0.157	0.108

r_s_: rho di Spearman. IL: interleukin; MCP-2: monocyte chemoattractant protein-2.

## Data Availability

The data presented in this study are available on request from the corresponding author.

## References

[1] Canzano P., Brambilla M., Porro B., Cosentino N., Tortorici E., Vicini S., Poggio P., Cascella A., Pengo M.F., Veglia F. (2021). Platelet and Endothelial Activation as Potential Mechanisms Behind the Thrombotic Complications of COVID-19 Patients. JACC Basic Transl. Sci..

[2] Jafarzadeh A., Chauhan P., Saha B., Jafarzadeh S., Nemati M. (2020). Contribution of Monocytes and Macrophages to the Local Tissue Inflammation and Cytokine Storm in COVID-19: Lessons from SARS and MERS, and Potential Therapeutic Interventions. Life Sci..

[3] Poissy J., Goutay J., Caplan M., Parmentier E., Duburcq T., Lassalle F., Jeanpierre E., Rauch A., Labreuche J., Susen S. (2020). Pulmonary Embolism in Patients with COVID-19: Awareness of an increased prevalence. Circulation.

[4] Comer S.P., Cullivan S., Szklanna P.B., Weiss L., Cullen S., Kelliher S., Smolenski A., Murphy C., Altaie H., Curran J. (2021). COVID-19 Induces a Hyperactive Phenotype in Circulating Platelets. PLoS Biol..

[5] Jamilloux Y., Henry T., Belot A., Viel S., Fauter M., El Jammal T., Walzer T., François B., Sève P. (2020). Should We Stimulate or Suppress Immune Responses in COVID-19? Cytokine and anti-cytokine interventions. Autoimmun. Rev..

[6] Varga Z., Flammer A.J., Streiger P., Haberecker M., Andermatt R., Zinkernagel A.S., Mehra M.R., Schuepbach R.A., Ruschitzka F., Moch H. (2020). Endothelial Cell Infection and Endothelitis in COVID-19. Lancet.

[7] E Fox S., Akmatbekov A., Harbert J.L., Li G., Brown J.Q., Heide R.S.V. (2020). Pulmonary and Cardiac Pathology in African American Patients with COVID-19: An Autopsy Series from New Orleans. Lancet Respir. Med..

[8] Ackermann M., Verleden S., Kuehnel M., Haverich A., Welte T., Laenger F., Vanstapel A., Werlein C., Stark H., Tzankov A. (2020). Pulmonary Vascular Endothelialitis, Thrombosis, and Angiogenesis in Covid-19. N. Engl. J. Med..

[9] Gelzo M., Cacciapuoti S., Pinchera B., De Rosa A., Cernera G., Scialò F., Mormile M., Fabbrocini G., Parrella R., Gentile I. (2021). Prognostic Role of Neutrophil to Lymphocyte Ratio in COVID-19 Patients: Still Valid in Patients That Had Started Therapy?. Front. Public Heal..

[10] Zhang X., Chen L., Dang W.Q., Cao M.F., Xiao J.F., Lv S.Q., Jiang W.J., Yao X.H., Lu H.M., Miao J.Y. (2020). CCL8 secreted by Tumor-Associated Macrophages Promotes Invasion and Stemness of Glioblastoma Cells Via ERK1/2 Signaling. Lab. Invest..

[11] Asano K., Takahashi N., Ushiki M., Monya M., Aihara F., Kuboki E., Moriyama S., Iida M., Kitamura H., Qiu C.-H. (2015). Intestinal CD169+ Macrophages Initiate Mucosal Inflammation by Secreting CCL8 that Recruits Inflammatory Monocytes. Nat. Commun..

[12] Cheung C.Y., Poon L.L., Ng I.H., Luk W., Sia S.F., Wu M.H., Chan K.H., Yuen K.Y., Gordon S., Guan Y. (2005). Cyto-kine Responses in Severe Acute Respiratory Syndrome Coronavirus-Infected Macrophages In Vitro: Possible Relevance To patho-Genesis. J. Virol..

[13] Chevrier S., Zurbuchen Y., Cervia C., Adamo S., Raeber M.E., de Souza N., Sivapatham S., Jacobs A., Bachli E., Rudiger A. (2020). A Distinct Innate Immune Signature Marks Progression from Mild to Severe COVID-19. Cell Rep. Med..

[14] Barthel S.R., Gavino J.D., Descheny L., Dimitroff C.J. (2007). Targeting Selectins and Selectin Ligands in Inflammation and Cancer. Expert Opin. Ther. Targets.

[15] Guo L., Sun G., Wang G., Ning W., Zhao K. (2014). Soluble P-selectin Promotes Acute Myocardial Infarction Onset But Not Severity. Mol. Med. Rep..

[16] Panicker S.R., Mehta-D’souza P., Zhang N., Klopocki A.G., Shao B., McEver R.P. (2017). Circulating Soluble P-selectin Must Di-merize to Promote Inflammation and Coagulation in Mice. Blood.

[17] Grobler C., Maphumulo S., Grobbelaar L., Bredenkamp J., Laubscher G., Lourens P., Steenkamp J., Kell D., Pretorius E. (2020). Covid-19: The Rollercoaster of Fibrin(Ogen), D-Dimer, Von Willebrand Factor, P-Selectin and Their Interactions with Endothelial Cells, Platelets and Erythrocytes. Int. J. Mol. Sci..

[18] Binder F.P.C., Ernst B. (2011). E- and P-selectin: Differences, Similarities and Implications for the Design of P-selectin Antagonists. Chim. Int. J. Chem..

[19] Karsli E., Sabirli R., Altintas E., Canacik O., Sabirli G.T., Kaymaz B., Özgür K., Koseler A. (2021). Soluble P-selectin as a Potential Diagnostic and Prognostic Biomarker for COVID-19 Disease: A Case-Control Study. Life Sci..

[20] Venter C., Bezuidenhout J., Laubscher G., Lourens P., Steenkamp J., Kell D., Pretorius E. (2020). Erythrocyte, Platelet, Serum Ferritin, and P-Selectin Pathophysiology Implicated In Severe Hypercoagulation and Vascular Complications in COVID-19. Int. J. Mol. Sci..

[21] Roldán V., Marín F., Lip G.Y.H., Blann A.D. (2003). Soluble E-selectin in Cardiovascular Disease and Its Risk Factors. Thromb. Haemost..

[22] Smadja D.M., Guerin C.L., Chocron R., Yatim N., Boussier J., Gendron N., Khider L., Hadjadj J., Goudot G., Debuc B. (2020). Angiopoietin-2 as a Marker of Endothelial Activation Is a Good Predictor Factor for Intensive Care Unit Admission of COVID-19 Patients. Angiogenesis.

[23] Blann A.D., Taberner D.A. (1995). A Reliable Marker of Endothelial Cell Dysfunction: Does It Exist?. Br. J. Haematol..

[24] Scavone C., Mascolo A., Rafaniello C., Sportiello L., Trama U., Zoccoli A., Bernardi F.F., Racagni G., Berrino L., Castaldo G. (2021). Therapeutic Strategies to Fight COVID-19: Which Is the Status Artis?. Br. J. Pharmacol..

[25] Zollo M., Ferrucci V., Izzo B., Quarantelli F., Di Domenico C., Comegna M., Paolillo C., Amato F., Siciliano R., Castaldo G. (2021). SARS-CoV_Subgenomic N (sgN) Transcripts in Oro-nasopharingeal Swabs Correlate With the Highest Viral Load, as evaluated by five different methods. Diagnostics.

[26] Cacciapuoti S., De Rosa A., Gelzo M., Megna M., Raia M., Pinchera B., Pontarelli A., Scotto R., Scala E., Scarano F. (2020). Immunocytometric Analysis of COVID Patients: A Contribution to Personalized Therapy?. Life Sci..

[27] von Cube M., Grodd M., Wolkewitz M., Hazard D., Wengenmayer T., Canet E., Lambert J. (2021). Harmonizing Heterogeneous Endpoints in Coronavirus Disease 2019 Trials Without Loss of Information. Crit. Care Med..

[28] WHO Working Group on the Clinical Characterization and Management of COVID-19 Infection (2020). A minimal Common Out-come Measure Set for COVID-19 Clinical Research. Lancet Infect. Dis..

[29] Ponthieux A., Herbeth B., Droesch S., Lambert D., Visvikis S. (2003). Age- and Sex-related Reference Values for Serum Adhesion Molecule Concentrations in Healthy Individuals: Intercellular Adhesion Molecule-1 and E-, P-, and L-Selectin. Clin. Chem..

[30] Elmoselhi H., Mansell H., Soliman M., Shoker A. (2016). Circulating Chemokine Ligand Levels Before and After Successful Kidney Transplantation. J. Inflamm..

[31] Van Damme J., Proost P., Lenaerts J.P., Opdenakker G. (1992). Structural and Functional Identification of Two Human, Tumor-Derived Monocyte Chemotactic Proteins (MCP-2 and MCP-3) Belonging to the Chemokine Family. J. Exp. Med..

[32] Lu L., Zhang H., Dauphars D.J., He Y.-W. (2021). A Potential Role of Interleukin 10 in COVID-19 Pathogenesis. Trends Immunol..

[33] Zhao Y., Quin L., Zhang P., Li K., Liang L., Sun J., Xu B., Dai Y., Li X., Zhang C. (2020). Longitudinal COVID-19 Profil-ing Associates IL-1RA and IL-10 with Disease Severity and RANTES with Mild Disease. JCI Insight.

[34] Han H., Ma Q., Li C., Liu R., Zhao L., Wang W., Zhang P., Liu X., Gao G., Liu F. (2020). Profiling Serum Cytokines in COVID-19 Patients Reveals IL-6 and IL-10 are Disease Severity Predictors. Emerg. Microbes Infect..

[35] Goshua G., Pine A.B., Meizlish M.L., Chang C.-H., Zhang H., Bahel P., Baluha A., Bar N., Bona R.D., Burns A.J. (2020). Endotheliopathy in COVID-19-Associated Coagulopathy: Evidence from a Single-Centre, Cross-sectional Study. Lancet Haematol..

[36] Neri T., Nieri D., Celi A. (2020). P-selectin Blockade in COVID-19-Related ARDS. Am. J. Physiol. Cell. Mol. Physiol..

[37] Liu Y., Xiang D., Gao F., Yao H., Ye Q., Wang Y. (2020). The inhibition of P-selectin Reduced Severe Acute Lung Injury in Immun-ocompromised Mice. Oxid. Med. Cell Longev..

[38] Littler A.J., Buckley C.D., Wordsworth P., Collins I., Martinson J., Simmons D.L. (1997). A distinct Profile of Six Soluble Adhesion Molecules (ICAM-1, ICAM-3, VCAM-1, E-selectin, L-selectin and P-selectin) in rheumatoid arthritis. Br. J. Rheumatol..

